# New insights into the association of air pollution and kidney diseases by tracing gold nanoparticles with inductively coupled plasma mass spectrometry

**DOI:** 10.1007/s00216-023-05105-8

**Published:** 2024-01-11

**Authors:** Souzana Angel, Lorna J. Eades, Gavin Sim, Alicja Czopek, Neeraj Dhaun, Petra Krystek, Mark R. Miller

**Affiliations:** 1 Centre for Cardiovascular Science, Queen’s Medical Research Institute, University of Edinburgh, 49 Little France Crescent, Edinburgh, EH16 4TJ UK; 2 School of Chemistry, The University of Edinburgh, Edinburgh, UK; 3 School of Geoscience, The University of Edinburgh, Edinburgh, UK; 4 Department of Chemistry and Biology, University of Siegen, Siegen, Germany; 5 Deltares, Utrecht, Netherlands

**Keywords:** Inductively coupled plasma mass spectrometry, Gold (Au), Nanoparticles, Kidney, Renal

## Abstract

Exposure to particles from air pollution has been associated with kidney disease; however, the underlying biological mechanisms are incompletely understood. Inhaled particles can gain access to the circulation and, depending on their size, pass into urine, raising the possibility that particles may also sequester in the kidney and directly alter renal function. This study optimised an inductively coupled plasma mass spectrometry (ICP-MS) method to investigate the size dependency of particle accumulation in the kidneys of mice following pulmonary instillation (0.8 mg in total over 4 weeks) to gold nanoparticles (2, 3–4, 7–8, 14 or 40 nm or saline control). Due to the smallest particle sizes being below the limit of detection in single particle mode, ICP-MS was operated in total quantification mode. Gold was detected in all matrices of interest (blood, urine and kidney) from animals treated with all sizes of gold nanoparticles, at orders of magnitude higher than the methodological limit of detection in biological matrices (0.013 ng/mL). A size-dependent effect was observed, with smaller particles leading to greater levels of accumulation in tissues. This study highlights the value of a robust and reliable method by ICP-MS to detect extremely low levels of gold in biological samples for indirect particle tracing. The finding that nano-sized particles translocate from the lung to the kidney may provide a biological explanation for the associations between air pollution and kidney disease.

**Graphical Abstract:**

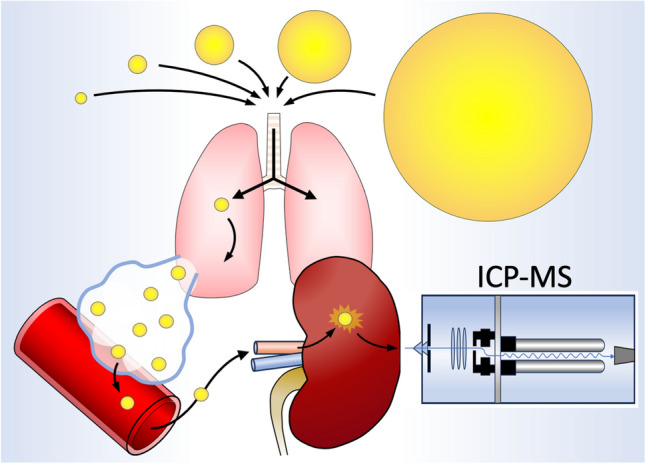

## Introduction

Air pollution is the greatest environmental risk factor for all-cause mortality globally, being linked to over 7 million premature deaths globally every year [1]. Exposure to air pollution has many wide-ranging adverse effects on health which go beyond that of the lung. Indeed, there is evidence linking air pollution with detrimental actions on all major organs of the body [2]. Recently, epidemiological studies have found associations between exposure to air pollution and kidney disease [3, 4], although the biological mechanisms underpinning these associations remain to be established.

While air pollution contains many different harmful constituents, it is the particulates in air pollution which are especially harmful to human health. Airborne particles originate from many different sources and as a result have a varied composition and size range. Ultrafine particles, such as those in diesel exhaust, are the smallest category of particulate matter (PM) with a diameter of 100 nm or less, with sizes that are smaller than many viruses. These particles are especially concerning due to their ability to penetrate deep into the alveoli of the lung and their high surface area-to-mass ratio with which to react with cells or carry harmful chemicals into the body [5]. Furthermore, there is growing evidence that ultrafine particles are small enough to cross from the lung into the circulation (“translocate”) and from thereon be carried to other organs of the body [6]. This pathway is a compelling explanation for why inhaled air pollution PM has detrimental effects throughout the body.

Detecting carbon-based nano-sized particles, like diesel exhaust particles, in the body is challenging due to the small size of the particles, the low levels of particles that translocate into the blood, their dilution in the blood and tissues, and that the cells of the body are high in carbon. Previously, we used gold nanoparticles as a model to address particle translocation in humans and animal models following inhalation [7]. Gold particles can be obtained in the same size range as diesel exhaust particles, and they are essentially inert so safe for human and animal exposure. Furthermore, very sensitive methods are available to aid their detection, and there is little background gold concentration in biological tissues naturally. By using these particles, we were able to demonstrate that inhaled nanoparticles crossed into the blood in humans and accumulated in inflamed arteries [7]. The translocation was size-dependent, and interestingly, the size thresholds were different for the blood (30–100 nm) than the urine (10–30 nm). The finding suggests that intermediate sized nanoparticles may sequester in the kidney. The primary particle size of a diesel exhaust particle is in the range of 10–80 nm; therefore, the accumulation of combustion-derived particles in the kidney could represent a means by which key sources of air pollution could promote kidney disease.

To confirm the uptake of nanoparticles into the kidney and further explore the size dependency, we performed repeated pulmonary exposure of different sizes of nanoparticles in mice. For this confirmation, a reliable and sensitive analytical method of ultra-trace detection of gold in biological matrices by inductively coupled plasma mass spectrometry (ICP-MS) was used as a means to indirectly measure gold nanoparticles in tissues [7, 8]. Within this study, progressive development of a multi-step analytical method was performed, resulting in a digestion method followed by ultra-trace quantification of gold with a modern ICP-MS type and optimised washout settings. Regarding to the identification and quantification by ICP-MS, the choice had to be made for external calibration with internal standard correction due to most of the administered nanoparticles being of a size range below 10 nm, which is below the limit of detection by the so-called single particle (sp) ICP-MS mode [9, 10].

Previous studies in rodents have demonstrated that nanoparticles cross from the lung into the blood to reach systemic organs (see [7, 11]) with a small number making measurements in the kidneys. However, to the best of our knowledge, only a single study [12] has made measurements in the kidney following pulmonary exposure of different sizes of nanoparticles. This study used only two sizes of particles and focused on differentials of the size of particle aggregates rather than primary (single) particles sizes. Here, we use a carefully selected size range of nanoparticles, with defined primary particle sizes (2, 3–4, 7–8, 14 and 40 nm) that span the main pore size of the kidney glomeruli (4–6 nm), to indirectly determine the size dependency of accumulation of nanoparticles in the kidney following pulmonary exposure.

## Materials and methods

### Materials and chemicals

Gold nanoparticles were custom synthesised by PlasmaChem GmbH, Germany (primary diameter sizes 2.2 and 40 nm) and Nanopartz, USA (3–4, 7–8 and 14 nm). Each size of nanoparticle was diluted to the lowest common concentration (2 mg/mL Au) in the supplier’s dispersant (particle-free citrate buffer). Nanoparticles were sonicated for 5 min at 100% prior to use. 

All chemicals used for sample pretreatment and mass spectrometry were of analytical grade or of high purity. Nitric acid (HNO_3_) and hydrochloric acid (HCl) of 99.9999% purity, or greater, were purchased from Thermo Fisher Scientific (UK), L-cysteine (Acros Organics, NJ, USA) and de-ionised (DI) water 18.2 MΩ produced with an Arium II system (Sartorious, Germany). The calibration standard solution of gold (Au), as well as a solution of the element rhodium (Rh) used as internal standard, was prepared using single-element stock solutions with a concentration of 1000 mg/mL (both PlasmaCAL grade, SPE Science, France). For quality control aspects on the relevant ultra-trace level, two reference samples “SEROnorm^TM^ Trace Elements in Whole blood L-1” and “SEROnorm^TM^ Trace Elements in Urine L-2” (both supplied by AB Scientific, UK) were included into the analytical procedure. Although the gold concentrations were not certified in these samples, they were used as reference matrices for additional control experiments. Isoflurane was obtained from Merial, UK.

### Exposure experiments

Experiments were performed in accordance with the United Kingdom Animals (Scientific Procedures) Act under a UK Home Office Licence. Adult mice (C57Bl6J; *N* = 72, *n* = 12 per group) were purchased at a starting age of 8–10 weeks (weight, 20–25 g) and given 1 week to acclimatise with standard rodent chow and water given *ad libitum*. Mice received repeated pulmonary exposure (800 μg in total, 50 μL of 2 mg/mL, twice weekly for 4 weeks) of gold nanoparticles (one of the following sizes, 2, 3–4, 7–8, 14 and 40 nm) or their particle-free dispersant as a control. Particle sizes were chosen to reflect the estimates of the main pore size of the glomeruli (~4–6 nm [13], see “Discussion”). Particles were delivered to the lung by aspiration instillation, as described in [14]. Briefly, mice were anaesthetised using isoflurane inhalation (~3 min), the tongue pulled forward with forceps, and a bolus suspension pipetted onto the oropharynx. The nares of the rodent were occluded using the forefinger and thumb, and the tongue held forward until total aspiration occurred. Animals were then placed back in their cage and monitored until return to normal behaviour (<5 min). Mice were humanely killed by exsanguination under terminal anaesthesia ~18 h after the final instillation of particles. Blood, kidneys and urine were collected and stored at −80°C until analysis.

### Analytical method

Approximately 500 mg of blood or whole kidneys (approximately 200 mg) was digested in 2 mL aqua regia (1:3 ratio of concentrated HNO_3_-to-concentrated HCl) at 80°C in disposable borosilicate tubes for 1 h on a block heater type (BT5D, Grant Instruments, UK). After the digestion was complete, 8 mL of ultrapure water was added to give a final volume of 10 mL. For each digestion batch, two reagent blanks were carried through the same procedure as the samples. Prior to analysis, the samples were further diluted by a factor of 10 with a solution of 0.01 % L-cysteine in 1 % v/v HCl to stabilise the gold during the analysis and to aid washout. The urine samples were directly diluted with 2 % (v/v) aqua regia by a factor of 10, left for 30 min and centrifuged for 5 min at 1400 rpm to remove the urine debris. The pre-treated samples were diluted in a solution of 0.01 % L-cysteine in 1 % v/v HCl by a factor of 10 immediately before analysis.

All samples were analysed for gold (Au) content using a multi quadrupole inductively coupled plasma mass spectrometer (multiquad-ICP-MS; type 8900, Agilent, USA). The instrumental setup and methodological settings of the ICP-MS are summarised in Table 1. Two external calibration curves for gold of the interference-free isotope ^197^Au were performed, a micro-calibration 0.001–0.5 ng/mL Au at the start of the analysis and a macro-calibration at the end of 0.01–2 ng/mL Au. Online addition and correction with an internal standard (200 ng/mL rhodium (Rh) with the measured isotope ^103^Rh) and the correction for the chemical blank were also applied. However, gold is a difficult element to analyse due to possible memory effects in the ICP-MS system, so a rigorous washout protocol was developed and tested prior to the analysis. This protocol includes the following optimisation steps: (1) selection and influence of used and cleaned *versus* new single-use digestion tubes on the background concentration of Au and (2) testing of different acids and solutions for reducing the background concentration of Au in blanks and washing solutions. It was concluded that new single-use tubes must be used for keeping the gold background concentration reliable and low. Additionally, sample dilution in 0.01% L-cysteine/1% HCl, introducing a blank check after each three samples and an improved washout protocol were applied during the measurement series to the pre-treated blood, kidney and urine samples. These steps were found to reduce the background and inter-sample carry over to <0.030 ng/mL Au for samples which were <1 ng/mL Au. Any samples that were analysed after a sample reporting >1 ng/mL in the digest were repeated to ensure carryover had not impacted the data. For the method validation, the methodological limit of detection (LoD) and limit of quantification (LoQ) were determined under reproducibility circumstances and in the blank (LoB) as well as in the three matrices of interest (blood, kidney and urine). As the concentration of Au is given a “<value” on the certificate, the SEROnorm^TM^ samples were used to check for blanks and also for the determination of the trueness by spike recovery using a spiked concentration of 0.5 ng/mL Au. The variation within and between analysis batches were determined by duplicate and triplicate measurements of selected samples.

**Table 1:** Instrumental setup and methodological settings of the ICP-MS

Parameter	Setting
Nebuliser	Micro-mist nebuliser by peristaltic pump at a rate of approximately 1.0 mL min^−1^
Spray chamber	Scott double pass (quartz)
Sampler and skimmer cone	Nickel
Take up time	1.5 min
Washout time	2 min
RF power	1550 W
RF matching voltage	1.8 V
Plasma gas flow	15 L min^−1^ Ar
Auxilary gas flow	0.9 L min^−1^ Ar
Nebuliser gas flow	1.03 L min^−1^ Ar
Measured isotope (internal standard isotope)	^197^Au (^103^Rh)
Operation mode	Single quad mode
Methodological settings	Fully quantitation mode with 1 point per mass unit and integration time of 0.1 ms; number of replicates: 3

## Results and discussion

With the optimised settings on the ICP-MS and the application of external calibration in two concentration ranges (always with internal standard correction), a dedicated analytical method validation was carried out for the quantification of traces of gold in various biological samples. This methodological selection is the most suitable approach for reliability and general time consumption when using a large batch series under operational conditions. The results of the most relevant performance characteristics are summarised in Table 2. The developed methodological limit of detection (LoD) and limit of quantification (LoQ) were determined under reproducibility circumstances for the three matrices of interest: blood, kidney and urine. In addition, the limit of detection was determined with the blank, which represents an instrumental limit of detection for the chosen settings. All results of the different limits of detection are very similar, and a clear trend can be recognised related to the complexity of the matrix, with blood having the greatest complexity. Additional preliminary experiments found that digestion blanks of around 0.2–0.3 ng/mL Au were obtained, which was too close to the anticipated concentrations for the lowest sized nanoparticle doses in tissues. Thorough testing of all the acids and reagents concluded that this was not the major contamination source, giving ranges of 0.001–0.012 ng/mL Au. The most significant improvement was achieved by switching to new borosilicate test tubes, with digestion blanks dropping to below 0.10 ng/mL Au. Further improvements were made by cleaning the lenses/glassware and using new sample and skimmer cones prior to first sample batch. Significant improvements were made by diluting samples in 2% v/v HCl and 0.01 % v/v L-cysteine which aided stability and washout. L-cysteine was also added to the wash cycle. The SEROnorm^TM^ reference samples were used to check the performance for the lowest gold levels are listed as <0.002 and <0.005 ng/mL Au for Whole Blood L-1 and Urine L-2, respectively; this was confirmed by several analyses. These SEROnorm^TM^ samples of the matrices blood and urine were also used for spike recovery experiments as well as a (blank) kidney sample; see Table 2. The recovery of the spiked concentrations of gold (Au) was >90%. This fulfils the purpose of using the developed method for further analysis of biological samples, while strict washout procedure had to be followed (see Table 1) to avoid possible memory effects and cross-contaminations. The variation within and between analysis batches is shown in Table 2.

**Table 2:** Performance characteristics of the developed analytical method

Performance characteristic	Results per (digested) matrix
Instrumental/blank limit of detection (LoB)	Blank solution (*n* = 7), (0.004 ± 0.002) ng/mL Au
Methodological limit of detection (LoD)	Biological matrices (*n* = 6), 0.005 ng/mL Au Blood (*n* = 2), 0.007 ng/mL Au Kidney digest (*n* = 2), 0.005 ng/mL Au Urine (*n* = 2), 0.005 ng/mL Au
Methodological limit of quantification (LoQ)	Biological matrices (*n* = 6), 0.013 ng/mL Au Blood (*n* = 2), 0.017 ng/mL Au Kidney digest (*n* = 2), 0.012 ng/mL Au Urine (*n* = 2), 0.0011 ng/mL Au
Trueness by spike recovery	Blood (*n* = 1), 90% Kidney (*n* = 1), 93% Urine (*n* = 1), 90%
Reproducibility within analysis batches	Blood (*n* = 3), SD 0.001–0.007 ng/mL Au Kidney (*n* = 3), SD 0.001–0.005 ng/mL Au Blood (*n* = 2), SD 0.005–0.028 ng/mL Au Urine (*n* = 2), SD 0.001–1.5 ng/mL Au
Reproducibility between analysis batches	Blood (*n* = 3), SD 0.062 ng/mL Au Kidney (*n* = 3), SD 0.018–0.068 ng/mL Au Urine (*n* = 2), SD 0.001 ng/mL Au

**Fig. 1:**
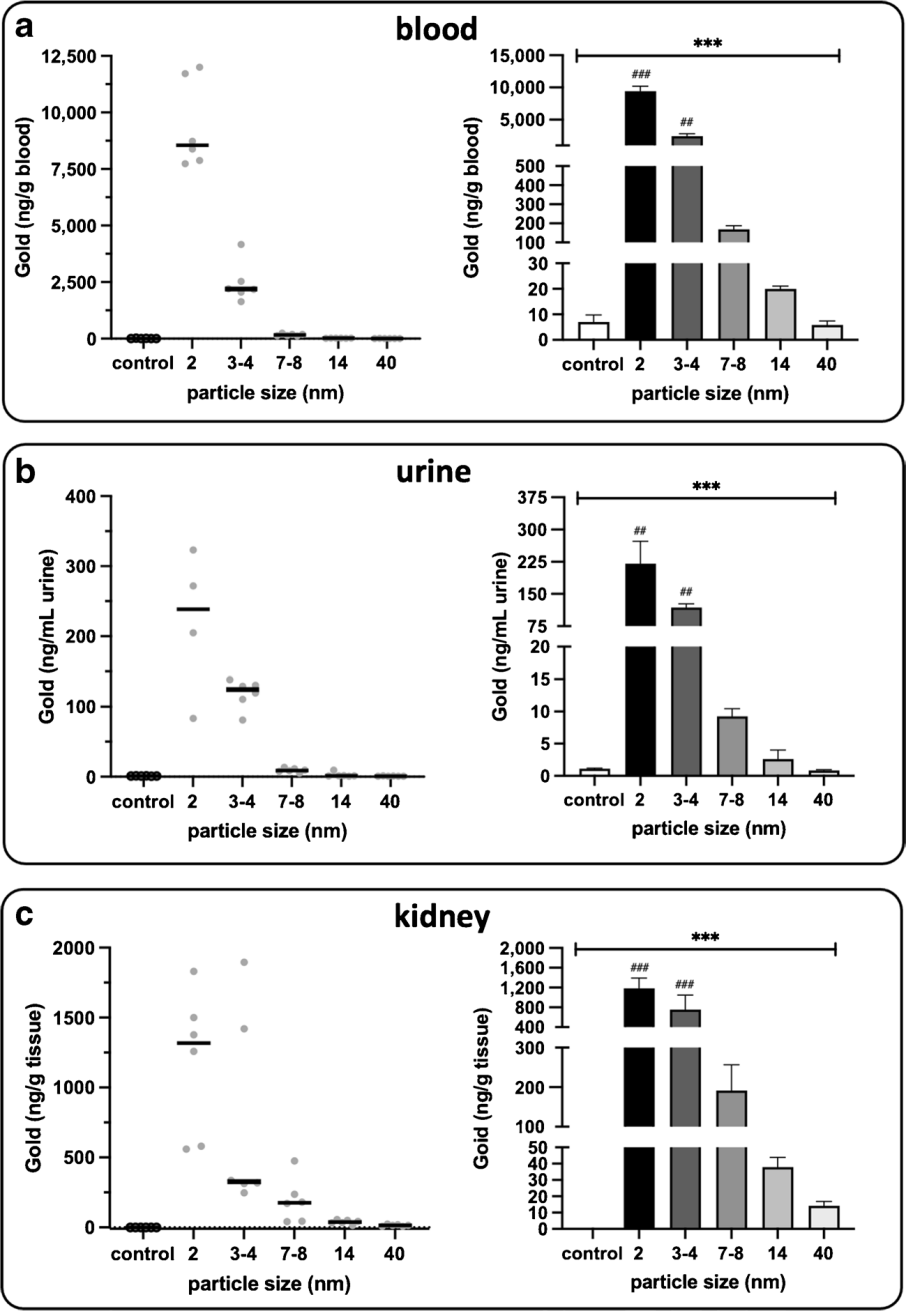
The levels of gold in tissue samples from mice following pulmonary instillation of gold nanoparticles. **a** Blood, **b** urine, **c** kidney. The left panels show individual points on a continuous scale. The right panels show means ± SEM (*n* = 6 for all, except 2 nm in urine where *n* = 4). ***P < 0.001; Kruskal–Wallis test comparing all groups, with Dunn’s multiple comparisons post-hoc test (^##^*P* < 0.01, ^###^*P* < 0.001compared to control)

Gold was detected in the blood of animals in a manner that was dependent of the size of particles (P<0.001); the smaller the particles administered, the greater the levels of gold in the blood (Fig. 1a). Gold was detected in the urine at lower concentrations but with a similar size dependency (Fig. 1b). Gold was also present in the kidney (Fig. 1c). In all tissues, detected gold levels of 2 nm and 3–4 nm gold particles were significantly greater than the control samples (vehicle control-treated animals) following adjustment for multiple comparisons (*P*<0.01).

A number of pathways have been proposed by which inhaled pollutants could have effects on organ systems beyond the lung. These include an induction of pulmonary inflammation which causes the release of inflammatory mediators into the circulation, and the activation of alveolar sensory receptors leading to changes in neural function that influence systemic organs through neural innervation of those organs or changes in the release of endocrine messengers [5]. Early in the twenty-first century, it was proposed that especially small particles were able to cross the alveolar barrier and access the circulation allowing them to directly interact with systemic organs [15]. The work of Kreyling et al. made considerable advances in this field, demonstrating that a range of labelled particles could access the circulation to reach other organs, exploring dependency factors such as particle size [12], charge [16] and adsorbed protein corona [17]. While the concentration of particles entering the circulation is small (<1%; see [11]), the biological consequences of translocated particles could occur through prolonged accumulation (e.g. over many years’ worth of exposure to air pollution) or if particles accumulate in susceptible regions of tissue, e.g. areas of disease [7].

Studies in rodents have demonstrated that particles from the lung can transit to the kidney (e.g. [18, 19]). A previous study has demonstrated that, following pulmonary exposure, particle aggregates show different levels of accumulation in the kidney depending on the size of the aggregate [12]. The current study aimed to build on these findings to explore the size dependency of accumulation in the kidney, specifically focusing on primary (single) particle sizes across the range of pore sizes in the kidney. The glomeruli of the kidney plays the key role in filtering particles from the blood. Estimates of pore size vary with different methodology, ranging from 3-26 nm [20, 21]; however, most estimates suggest that the majority of pores have a median diameter of ~4–6 nm, with a second subset of pores in the region of 9–14 nm [13, 21, 22]. Although our study did not set out to measure glomerular pore size, our results concur with these size estimates, with far greater numbers of sub-7 nm particles passing through the kidney into the urine, but with smaller amounts of larger nanoparticles (14 nm) also present in the urine. Physiologically, size alone is not the only determinant of glomeruli filtering, as molecular shape, charge and changes in biological permeability (e.g. from inflammation or disease) will also impact the passage of substances through the kidney.

The findings support the notion, that while excretion to the urine is a method of clearing translocated particles from the blood, particulates still accumulate in the renal tissue. Due to the dominance of small particles in the blood, a similar size dependency was also found in the kidney. The results suggest that small particulates have means to penetrate into renal tissue as opposed to accumulation of only those particles which are unable to penetrate through the pores of the glomeruli. Future studies using intravenous delivery of equivalent blood concentrations of each size of nanoparticle could provide insight into the size specificity of handling of blood-borne nanoparticles by the kidney.

The accumulation of particles in the kidney provides a potential biological mechanism for epidemiological associations between exposure to particulate air pollution and chronic kidney disease. In humans, exposure to PM_2.5_ (particulate matter with a diameter of 2.5 µm or less; the smallest size fraction of airborne particulates measured by stationary air pollution monitoring networks) impairs renal function: decreasing glomerular filtration rate, increasing serum creatinine, inducing albuminuria and increasing levels of kidney injury molecule-1 (KIM-1) [4]. Animal studies have explored this mechanism of impairment further by investigating the effect of diesel exhaust particles (DEP), a common urban air pollutant that is rich in nano-sized particles. These studies have shown that pulmonary exposure to DEP increases urinary N-acetyl-beta-glucosaminidase (NAG) (a marker of renal tubular dysfunction) and reduces renal levels of the antioxidant glutathione [23, 24]. The pro-oxidative effects of DEP were greater in animals with pre-existing kidney injury [23, 25, 26]. There is now a need for studies to address which sources, constituents and sizes of particulate air pollutants are likely to be particular toxic to the kidney, as these studies would provide insight into which individuals may be at greater risk of harm and help guide effective and targeted interventional measures to reduce exposure.

## Conclusions

Our study confirms that inhaled particles pass into the blood and accumulate in systemic organs. Accumulation of translocated particles into the kidney represents a potential mechanism by which air pollution could promote kidney disease. The application of a strict sample pretreatment procedure followed by the analysis by ICP-MS, with careful protocols to avoid contamination as well as to achieve system stability and reliability, represents a valuable tool for exploring the biodistribution of gold traces related to nanoparticles and builds a relevant multi-disciplinary research field.

## Data Availability

The datasets generated during and/or analysed during the current study are available from the corresponding author on reasonable request.
